# A chasm: Consequences of poor collaboration between health and education in paediatric cerebral palsy care in Johannesburg

**DOI:** 10.4102/sajcd.v68i1.817

**Published:** 2021-08-19

**Authors:** Martha Lydall, Berna Gerber

**Affiliations:** 1 Department of Speech-Language and Hearing Therapy, Faculty of Medicine and Health Sciences, Stellenbosch University, Stellenbosch, South Africa; 2 Speech Therapy Department, Forest Town School, Johannesburg, South Africa

**Keywords:** perceptions, rehabilitation, disability, referral, education, procedure, South Africa

## Abstract

**Background:**

Nearly 20 years since the establishment of the National Rehabilitation Policy, strides have been made within the health and education sectors to improve accessibility to rehabilitation services as well as the quality of life of children with cerebral palsy (CP). Shortfalls, however, still exist in implementing the policy. An in-depth study into the implementation of policy would be beneficial in identifying and understanding the shortfalls of the rehabilitation process.

**Objectives:**

To investigate the perceptions of Speech-Language Therapists (SLTs) working in the Gauteng Department of Health (GDH) and Gauteng Department of Education (GDE), in Johannesburg Region A, about systemic strengths and weaknesses surrounding the service delivery for children with CP, from birth to 6 years.

**Method:**

A qualitative study was conducted. Thirty-one (31) SLTs working in public hospitals, clinics and schools for Learners with Special Educational Needs participated in eight focus group interviews. Interviews were audio-recorded for transcription and subsequent thematic analysis.

**Results:**

The participants reported a lack of resources and knowledge that contributed to a perceived chasm between the GDH and GDE, resulting in fragmented and uncoordinated service delivery for children with CP leaving the health system and entering the education system.

**Conclusion:**

The results suggest that a cohesive plan should be formulated to bridge the perceived chasm between GDH and GDE in the referral process of children with CP from the health setting, into the school environment. This may facilitate communication, collaboration, education, as well as resource-sharing between the departments. Rehabilitation professionals should actively participate in such planning processes.

## Introduction

The South African Department of Social Development (2009) states that there remain fragmented and unequal services for children with disabilities in the country. Government departments are uncoordinated and act in isolation. Early identification of disabilities and tracking of progress through early childhood to school-going age remain systematically deficient (Department of Social Development, 2009). This is the situation, despite progress made in legislative and policy reform. Furthermore, as noted by Rule, Lorenzo and Wolmarans (2006), many children are receiving formal rehabilitation for the first time when they enter the formal schooling system. Given the socio-economic environment of the country, despite policy dictating that a child’s formal education begins at 6 years of age, this can often be later (Munnik & Smith, 2019). Speech-Language Therapists (SLTs) working in the public sector, given their interaction with the systems and procedures in place with regard to the management of children with cerebral palsy (CP), can provide valuable insight into the effectiveness of policies. An in-depth investigation into the perceptions of such SLTs could assist in identifying deficiencies in existing policies as well as suggestions to rectify these. This could facilitate improved service delivery, and cohesion and collaboration between sectors.

Cerebral palsy is a neurodevelopmental disorder that affects movement and posture because of nonprogressive damage to the immature brain, thus affecting the child throughout his or her life (Rosenbaum, Paneth, Leviton, Goldstein, & Bax, 2006). As a child develops into an adult, the severity of the disability and functional level of the individual changes. The functional level achieved as an adult is entirely dependent on the levels of function that the individual was able to reach as a child. Achieving the best possible outcomes is facilitated through continuous rehabilitation to achieve maximum potential and prevent functional deterioration from occurring (Vos et al., 2013).

For a long time in South Africa, continuous rehabilitation had not been provided to children with disabilities. Therefore, a need arose to develop a strategy that addresses the neglect of paediatric rehabilitative services. This gave rise to the *Integrated National Disability Strategy* that was established in 1997 by the Office of the Deputy President of South Africa (1997). For optimal functional levels to be reached and maintained within children with disabilities, by means of rehabilitation, the *Integrated National Disability Strategy* proposed a National Rehabilitation Policy (NRP) (Office of the Deputy President, 1997). The NRP was finalised and published in 2001.

Simultaneously in 2001, the Department of Education (DoE) issued a framework policy document called White Paper 6 (WP6): *Special Needs Education, Building an Inclusive Education and Training System* (Department of Education, 2001). This policy document set out the government’s proposal for future legislation regarding special needs education. The policy acknowledged that all children and youth can learn and those requiring remediation to cope with educational demands should be provided with support. Furthermore, the policy aimed to acknowledge the education structures, systems and learning methodologies required to meet the needs of all learners (Department of Education, 2001).

In 2013, the South African Department of Health recognised that to address the health inequalities experienced by persons with disabilities in our society, a transformation of the current South African health system had to occur. Hence, the *Framework and Strategy for Disability and Rehabilitation Services in South Africa* (National Department of Health, 2016) was compiled. This framework aims to reconfigure rehabilitation as an integral part of health services across all programmes, within all levels of care. It aims to increase access, equitability, inclusivity and participation for the population living with disabilities within their communities (National Department of Health, 2016). The framework recognises that rehabilitation services are a vital link between medical treatment and the transformation of a person’s restored capacity into a productive and health-promoting social and economic life (National Department of Health, 2016). Furthermore, it highlights a number of challenges that complicate the effectiveness of rehabilitation services within the population of children with CP in South Africa such as poor collaboration between sectors, and poor knowledge in the rehabilitation chain regarding the specific needs and challenges of persons with disabilities (National Department of Health, 2016). Furthermore, the framework states that appropriate referral pathways create access to suitable care and these must be aligned with departmental policy.

Louw et al. (2018) stated that access to effective rehabilitation is a basic human right and that rehabilitation has the ability to economically optimise health outcomes and the overall quality of life. However, the 20 rehabilitation professionals (physiotherapists, occupational therapists, SLTs, podiatrists, rehabilitation managers or directors) who participated in Louw et al.’s (2018) study stated that, despite the South African government’s efforts to re-engineer public healthcare, rehabilitation services at primary healthcare level have not improved (Louw et al., 2018).

A study on the experiences and challenges faced by rehabilitation community service therapists within the South African Public Health Care (PHC) system (Ned, Cloete, & Mji, 2017) revealed that the conditions at community healthcare level are particularly difficult as there is a scarcity of professionals, failures in resource allocations, as well as overcrowding. A study conducted by Khoza-Shangase and Mophosho (2018) supported this finding, specifically stating that there is a shortage of SLTs working in the public health sector. When referral happens, in some instances, there are no human resources available to receive these referrals at the community level (Hussey, MacLachlan, & Mji, 2017). Premature discharge from tertiary and secondary levels of care, with referral into the primary level of care, often creates problems with carryover of care because of a lack of follow-up between institutions. Collaboration between the various levels of care would result in comprehensive insight amongst all stakeholders in the care chain into contextual issues of disability and further promote investigations by these stakeholders into health systems (Ned et al., 2017).

Nearly 20 years since the development of the NRP and WP6, strides have been made in both the health and education sectors to implement policy in order to improve accessibility to all rehabilitation services and thereby improve the quality of life of children with disabilities (Department of Basic Education, 2015). Yet, anecdotal evidence as well as research conducted by the National Department of Health (2016), Louw et al. (2018) and Ned et al. (2017) suggests that limited collaboration exists between these sectors, resulting in continued fragmented and uncoordinated service delivery.

The main aim of this study was to investigate the perceptions of SLTs working in the public health and education sectors in the greater Johannesburg region, about institutional strengths and weaknesses within service delivery for children with CP, from birth to 6 years of age. The objectives were as follows:

- To report on the SLTs’ perceptions about the availability of services for children with CP in the public health and education sectors.
- To describe the carryover of rehabilitation of children with CP between hospitals, clinics and schools.
- To identify the strengths and weaknesses of the referral pathway for children with CP in the public sector.

## Research methods and design

### Study design

A qualitative research design was used. Qualitative research methods can provide detailed descriptions of the insights of individuals, useful quotes that bring realism to applied research, as well as information about how different healthcare settings operate (Taylor, Bogdan, & DeVault, 2015).

### Setting

Participants were recruited from public health and education institutions in Region A of Johannesburg. Johannesburg was selected for this study because of its cosmopolitan nature. Two central hospitals are located in this region. Furthermore, the first author works as an SLT in this region and had professional motivation to investigate the research objectives as they apply to this particular geographic area.

### Study population and sampling strategy

A purposive sampling strategy was used for this study. Qualified SLTs working with children with CP in the public health (hospitals and Community Health Centres [CHCs]) and education (Learners with Special Educational Needs [LSEN] schools) sectors were asked to participate (Table 1). Participants ranged in age from 23 to 65 years, with varying levels of experience ranging from less than 1 year working experience to 43 years of experience. Speech-Language Therapists were included based on the following criteria:

- Minimum of a 4-year degree in Speech-Language Therapy or Speech-Language Therapy and Audiology.
- Registered with the Health Professions Council of South Africa.
- Working in hospitals (tertiary, central and district hospitals), CHCs and LSEN school settings in the public sector, in Region A of Johannesburg, Gauteng province.
- Therapists specifically working with children with CP from birth to 6 years as part of their current caseload, or within their last 6 months.

**TABLE 1:** *Number of* speech-language therapists *who participated in the study*.

Gauteng region A – Health and education facilities	Number of facilities in the region	Number of facilities that participated	Total number of SLTs working in the institutions	Total number of SLTs who met the inclusion criteria	Total number of SLTs who participated†
Central hospitals	2	2	48	9	8
Tertiary hospitals	4	2	13	7	4
District hospitals	2	1	4	2	2
CHCs in Johannesburg metro rehab‡	9	9	8	8	8
LSEN schools§	3	3	10	10	9
Column totals	20	17	83	36	31

SLT, Speech-Language Therapists; LSEN, Learners with Special Educational Needs; CHC, Community Health Centres.

† , Speech-Language Therapists who met the inclusion criteria and gave informed consent.

‡ , Primary Healthcare Facilities include clinics and Community Health Centres (CHCs) of which only CHCs were included in the study (Gauteng Provincial Government, 2018).

§ , The schools for Learners with Special Educational Needs (LSEN) in the public sector of the region, who admit children with cerebral palsy presenting with Mild and Moderate Intellectual Disabilities (MID) (Gauteng Department of Education, 2019) were included in the study.

All participants met the inclusion criteria. Three therapists working in the hospitals and five working in the CHCs were completing their year of compulsory community service. Therapists with more than 10 years’ working experience with children with CP were based at the LSEN schools. This level of experience did not exist in the hospital and clinic settings.

### Data collection methods

An interview guide was used to conduct semi-structured interviews with the participants.

#### Ensuring trustworthiness

The trustworthiness of a study refers to ensuring credibility, confirmability, dependability, as well as transferability (Elo et al., 2014). Credibility was achieved through the pilot study and member checking processes, confirmability through consultation with colleagues and peers, dependability through the use of purposive sampling, and transferability by providing an accurate and honest description of the research process, context and participants.

#### Pilot study

A pilot study was conducted to evaluate the feasibility of the study, specifically the effectiveness and appropriateness of the interview guide. The guide was developed to address the research objectives after a comprehensive literature review was undertaken.

#### Focus groups and depth-interview

The interviews were conducted in the hospitals, CHCs and school settings where there was a team of SLTs working with children with CP. They were conducted at the therapists’ place of work. The therapists working at the CHCs all travelled to a central CHC to participate in the focus group. The number of participants in each focus group ranged from three to eight. All interviews were conducted in a space free from external noise. The interviews lasted approximately 30 min – 60 min each. Eight focus groups were conducted in total, and one individual depth-interview, where there was only one therapist employed at the institution. Four of the focus groups and the individual depth-interview were conducted at the hospitals, one focus group was conducted at a CHC, and three were conducted at the schools.

#### Audio recordings

Each participant was required to sign consent for the interview to be audio-recorded for later transcription and analysis. Author A listened to the recordings and transcribed all the interviews verbatim. The abbreviations ‘HT’ (Hospital Therapist), ‘CT’ (Clinic Therapist) and ‘ST’ (School Therapist) were used to protect the participants’ identities.

### Data analysis

**FIGURE 1:**
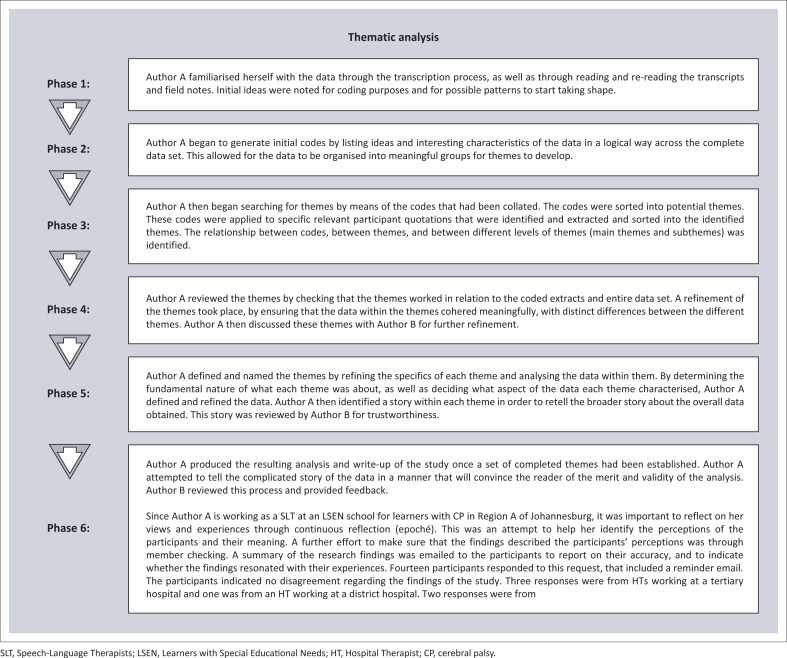
Thematic analysis as proposed by Braun and Clarke (2006).

Thematic analysis using the approach proposed by Braun and Clarke (2006) (Figure 1) was used in the analysis of the data.

### Ethical considerations

An application was made to Stellenbosch University’s Health Research Ethics Committee (HREC) for permission to proceed with the research. The permission was granted in June 2019 with the HREC Reference number: S19/05/093. A further request to conduct research at the specified hospitals, CHCs and LSEN schools was sought and granted. An information sheet and informed consent form was given, in person, to each potential participant, explaining the aim of the research and the research procedure. Only participants who provided informed consent were interviewed.

## Findings

**FIGURE 2:**
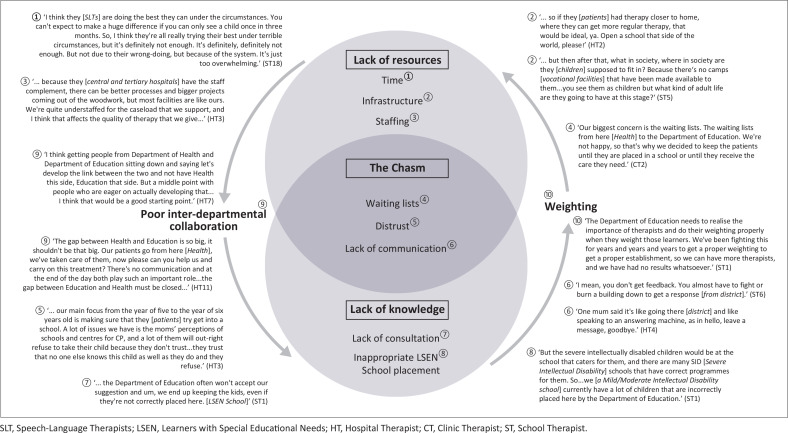
Visual representation of research data obtained through thematic analysis.

The findings are summarised in Figure 2. Three major themes emerged, highlighting institutional shortcomings within service delivery for children with CP. The circles represent the relationship between the themes: the lack of resources, lack of knowledge, and the consequent ‘chasm’. The arrows represent the cyclic process of how a lack of knowledge together with weighting, a process of assigning values to different disabilities based on pre-determined formulas for the purposes of staffing allocation, entrenches a lack of resources whilst failures in interdepartmental collaboration perpetuate a lack of knowledge.

### Perceptions of a lack of resources

Time, infrastructure and adequate staffing are the resources that were identified by the participants as lacking in the public sector. High caseloads with low staff complements, accompanied by poor physical infrastructure and time constraints, resulted in increased frustration levels, low morale, and a sense of despair amongst the participants. The sense of defeat stemming from the inadequate current institutional facilities available for children with CP, such as the number of therapists in Department of Health (DoH), LSEN schools, training and stimulation centres (institutions that provide care and/or education for children with lower cognitive abilities), and the genuine concern about the lack of vocational institutions available for these children once they leave school, was palpable.

### Perceptions of a lack of knowledge

A strained relationship with the Gauteng Department of Education (GDE), as a bureaucratic collective, was communicated by most of the participants. The participants perceived a general lack of understanding by the administrators at the GDE as to the nature of CP and what therapeutic interventions are required to ensure the best possible outcomes for this population. Participants working in school settings felt that the GDE did not focus on the best interest of the children with CP during the admissions process. The perception of the participants was that the GDE did not appropriately allocate children to specific LSEN schools, given the children’s cognitive and physical level of functioning. This constituted an important aspect of the narrative. Participants conveyed dissatisfaction about several facets, namely a general lack of consultation from the GDE, a lack of urgency in LSEN school placement, inappropriate admissions into LSEN schools hampering the efficacy of the curriculum, inappropriate admissions into LSEN schools with weighting scores for learners and the negative consequences of this on staffing, and the lack of infrastructure. Participants working in the school setting reported that inappropriate weighting of learners in LSEN schools negatively affected the staffing at the school. They stated that if the schools did not appropriately admit learners with specific disabilities, they did not achieve the desired child-to-staff ratio, and this resulted in fewer allocations of therapists and teachers to schools.

### Perceptions of a chasm between the Department of Health and the Department of Education

A consistent theme that emerged from the data indicated the perception of a chasm between the Gauteng Department of Health (GDH) and the GDE. The participants reported that this perceived chasm resulted in a belief amongst caregivers that the ‘transfer into the next stage of life’ (HT3) will fail. Some participants stated that the caregivers experienced apprehension in leaving the hospital and/or clinic setting and entering the education system. The participants expressed that the fear of the unknown accompanied by the slow process, such as waiting lists at the district-level of the education sector, resulted in delayed procedures and reluctance and distrust of caregivers to move their children’s rehabilitation from the familiar healthcare sector. A further finding was that there was a desire in hospital, clinic as well as school-based participants to close this chasm that seems to exist between the two departments, by creating collaborative, interdepartmental systems. They were aware that improved communication between therapists during carryover procedures will improve handover of the child’s care.

## Discussion

Given that CP is a lifelong condition that requires ongoing intervention to assist the child and family to function in the most effective way possible (Levitt & Addison, 2019), it is paramount that governments provide adequate staffing and infrastructure within public hospitals, clinics and schools. Yet, the provision of specialised facilities with adequate staff for this population is an ongoing challenge in South Africa. Not all healthcare and educational facilities equally accommodate the varied needs of children with CP. Without sufficient and appropriate facilities, therapists are often unable to refer children to a facility where their specific needs will be met. Johannesburg, being home to the largest cosmopolitan population in the country (Statistics South Africa, 2016), is one of the most resourced regions in the country. The insights gained through this study into difficulties in departmental collaboration, communication and handover, are thus noteworthy.

The participants in our study reported that interinstitutional collaboration assists in the mitigation of therapeutic regression of each child that can occur because of handover from one institution to the next. Where given the opportunity, relationships between participants at different institutions in Gauteng Region A have been created, attesting to a sense of agency amongst these therapists. However, administrative hurdles in the provincial DoH and the DoE made it difficult for collaborative interinstitutional relationships to develop.

Each child with CP is unique and this highlights the importance on the referral process from one rehabilitation institution to the next. There was an overwhelming sense of frustration amongst participants in both the provincial health and education sectors that highlighted a desperate need to address the lack of communication between these two sectors. According to the National Department of Health’s *Framework and Strategy for Disability and Rehabilitation Services in South Africa* (National Department of Health, 2016), rehabilitation should be decentralised and begin as early as possible and should extend from community to tertiary and specialised rehabilitation levels. The frustration amongst the participants resonates with the findings of Watermeyer, Penn, Scott and Seabi (2019) regarding difficulties with communication in decentralised tuberculosis care. It seems that when communication between healthcare, education institutions and administrative entities fails, responsibility for communication and continuity of care is unduly shifted onto the patient or caregiver (Watermeyer et al., 2019). As reported by some of the participants, the therapists are often excluded from the school admissions process because of procedures that rely on the simplified classification of cognitive abilities of each child, from a limited group of medical professionals, signifying a medical model approach. Yet, the Department of Basic Education (2014) specifically states that the medical assessment on its own must not be used to make decisions about admission to a special school. The assessment should be conducted by all the relevant professionals. Without in-depth diagnostic assessments performed by rehabilitation professionals, it is unsurprising that inappropriate school placements for children with CP are common. The SLT’s specialised understanding of a child’s communication difficulties and other associated complications in the management of CP should not be overlooked in the referral process. Procedural review and reform at the GDE could be instrumental in removing barriers to interprofessional communication and ensuring that rehabilitative gains made at hospital and clinic-level are carried over reliably into the therapeutic environment at LSEN schools in the province. Furthermore, a consultative process with rehabilitation professionals, including SLTs, occupational therapists, physiotherapists, as well as educational psychologists working at LSEN schools, could substantially reduce the incidence of inappropriate admissions.

Failure to place children into schools where the existing resources and infrastructure are aligned with the specific needs of the child, placed enormous strain on the participants. Participants reported that feedback that suggested a particular school was not a suitable placement for specific children with CP was often met with resistance by the GDE. According to the guidelines of the Department of Basic Education (2014) appropriateness of placement should be reviewed annually or at least every 2 years. The reason why the GDE appears to act in a manner contrary to this departmental policy was not investigated in this study. The overall effect of this perceived lack of understanding seems to be that there is an inappropriate distribution of resources, a lack of direction in the establishment of new infrastructure, and the destruction of the existing cohesive elements within the system.

The participants perceived a gap in communication between the DoH and the DoE in the referral process of children with CP from the hospital or clinic setting, into the school environment.

The findings of this study suggest that a cohesive plan needs to be formulated and executed to bridge this chasm, and to facilitate communication, collaboration, education, as well as resource-sharing between the two departments. Bidirectional communication enabled by a cohesive plan may improve the referral pathway and result in improved handover and carryover of therapeutic strategies into appropriate LSEN schools, preventing children from ‘falling through the cracks’ (HT8). This will also assist caregivers in making the transition from the health setting into the education setting and thus improve their trust in the ‘system’ and individual institutions.

Therapists who deliver services to clients and families can and should be actively part of or even initiate and drive such planning. Rather than assuming a position of reliance on higher level administrative divisions for reforms and plans, rehabilitation professionals should confidently and assertively fulfil their advocacy role within the healthcare and education systems. The multidisciplinary rehabilitation team possess a wealth of knowledge and skills. Speech-Language Therapists are valuable as experts in communication and may develop, monitor and evaluate communication processes.

### Strengths and limitations

As qualitative research is focused on attaining in-depth understanding of the perceptions of participants, the number of participants is necessarily limited. The limited sample size of 31 participants forming eight focus groups was, however, representative of the region under investigation, as many therapists approached via the purposive sampling strategy agreed to participate (31 out of 36). This yielded good insight into the perceptions of the participants with data saturation being achieved, giving credence to the findings. However, conclusions about the perceptions, practices and procedures of therapists working in other parts of the province and country should not be drawn.

### Recommendations

As the GDE is responsible for placing children in schools, further research should be conducted at the administrative district level of the GDE to further investigate the perceived chasm between the provincial departments of health and education. With the insights obtained from such an investigation, further recommendations can be made for the implementation of improved communication procedures and strategies between sectors and institutions. This communication needs to be bidirectional and create opportunities for the GDE to inform the GDH of the services available in LSEN schools, and for the GDH to be able to share relevant referral information about a child, when assisting with school placement. This communication should be conducted in a manner that fosters trust.

Although the study was set out to investigate the (dis)continuity of speech-language therapy services for children with CP in the public sector specifically, the results highlighted that some of the contributing factors fall outside the direct purview of SLTs. Further research could include investigating the perceptions of the doctors making diagnoses and referring to the educational districts for LSEN school placement. Their perceptions of the various LSEN schools and motivations for recommending one school as opposed to another can further increase understanding and possible improvement of the referral process.

Lastly, further investigations similar to the current study should be conducted in other provinces, as well as with other health professionals such as physiotherapists and occupational therapists, to establish whether there are correlations with the findings of the current study.

## Conclusion

Cerebral palsy is a lifelong condition that requires ongoing intervention. It is therefore paramount that governments should provide adequate staffing and infrastructure within public hospitals, clinics and LSEN schools. Each child with CP is unique, which reinforces the importance of the referral process from one institution to the next. By far, the most prominent theme which emerged was the perceived chasm between the health and education sectors with regard to communication rehabilitation. Carryover of therapeutic goals and strategies within the referral pathway is negatively affected by a general lack of communication, collaboration and resource-sharing between the two departments. There is also little relationship between the SLTs working in the public health sector and those working in the education sector.
